# Predictions of Immunodominant Epitope Peptides From the AsaA Type VI Secretion System in Acinetobacter baumannii: A Computational Approach

**DOI:** 10.7759/cureus.59618

**Published:** 2024-05-04

**Authors:** Medha Ranjan, A. S. Smiline Girija, Vijayashree J Priyadharsini

**Affiliations:** 1 Department of Microbiology, Saveetha Dental College and Hospitals, Saveetha Institute of Medical and Technical Sciences, Saveetha University, Chennai, IND

**Keywords:** acinetobacter baumannii, health, epitopes, vaccine peptides, asaa, t6ss

## Abstract

Introduction

*Acinetobacter baumannii*, designated as a priority pathogen by the World Health Organization (WHO), is responsible for recalcitrant infections in immunocompromised patients. The type VI secretion system (T6SS) is a class of macromolecular secretion machines, contributing to its virulence. The aim of this study is thus to predict the immune-dominant epitope peptides from the Acinetobacter T6SS-associated protein of *A. baumannii* (AsaA).

Methods

AsaA protein retrieval from the bacteria was carried out using computational platforms and the evaluation of antigenicity and allergenicity was performed. The T-cell epitopes of major histocompatibility complex class II binders were identified followed by molecular docking of the immune-dominant epitopes with human leukocyte antigen alleles using the ClusterPro server (https://cluspro.org/help.php). Additionally, the B-cell epitopes were predicted.

Results

Immune-informatic analysis showed immune-dominant peptides in the most favored regions with promising interactions with HLA alleles DP, DQ, DR, and toll-like receptor showing high binding capacity.

Conclusion

In the present investigation, epitope 1 (LILFLIGNY) was found to be a promising candidate for the synthesis of vaccines. However, it requires further experimentation for its immunological memory and response.

## Introduction

*Acinetobacter baumannii* is a non-fermenting gram-negative pleomorphic bacteria and an important nosocomial pathogen causing recalcitrant infections [1]. This coccobacillus had been previously regarded as a pathogen of low priority; however, it has been found that it is an opportunistic pathogen causing systemic infections including septicemia due to its propensity of multi-drug resistance (MDR) [2]. *A. baumannii* is ubiquitous in nature. They can be recovered after enrichment culture is obtained virtually from soil or surface water. Increasing reports on its virulence and rapid acquisition of the property of MDR are often reported among the *A. baumannii *traits [3]. The World Health Organization has declared *A. baumannii* as a priority pathogen due to its resistance to the last resort of antibacterial drugs. It is been categorized under the ESKAPE (*Enterococcus faecium, Staphylococcus aureus, Klebsiella pneumonia, Acinetobacter baumannii, Pseudomonas aeruginosa, *and *Enterobacter *species) group of pathogens [4]. 

In *A. baumannii,* several virulence factors have been characterized both genotypically and phenotypically and they encompass the porins in the outer membrane, potent proteases, phospholipases, cell wall lipopolysaccharides (LPS), polysaccharides in the capsule, iron chelating systems, and secretion systems of virulent proteins [5]. The type VI secretion system (T6SS) is a recently described secretory machine used widely by gram-negative bacteria in order to target their eukaryotic and prokaryotic competitors [6]. These macromolecular secretion mechanisms facilitate the entry of various effector proteins into the target cells. This process, in turn, is linked to the anti-phagocytosis and anti-apoptotic functions of the cell and is highly associated with bacterial virulence [7]. The secretion system contains several proteins that form needle-like structures that further help the gram-negative bacteria for the translocation of its substrates. The T6SS is competent in mediating inter-bacterial competitions which aids the bacteria to survive in their natural habitat [8].

The Acinetobacter T6SS-associated protein in *A. baumannii *(AsaA) is known to be comprised of 20 genes, and the first gene in order is the AsaA which is highly specific to the genus. Not many experiment-based evidenced documents are reported for their specific contribution to the bacterium. In a study done on the AsaA homolog analysis in *Acinetobacter baylyi*, AsaA was attributed to the T6SS and a high genetic difference occurred in comparison with the traits of *A. baumannii* [9]. In another study, the AsaA gene is documented to be highly specific to *A. baumannii* and is also highly conserved contributing to the virulence of the bacterium [10].

Targeting the secretion systems would be thus a novel approach to alleviating the *A. baumannii *complications among hospitalized patients. In-silico-based computational platforms are widely employed tools to predict the immuno-dominant peptides that may be used to design the vaccine constructs [10-11]. The approach has been applied in many studies to predict the epitope peptides in *A. baumannii* for various virulent proteins. The objectives of the present study were thus framed to predict the peptide epitopes from the AsaA protein and to analyze its properties of antigenicity, stability, human leukocyte antigen (HLA) interactions, major histocompatibility complex (MHC) class restrictions, and B-cell antigenicity. The study aims to predict the immune-dominant epitope peptides from the AsaA protein in *A. baumannii *using an immune-informatics approach that may be chosen as a suitable vaccine candidate.

## Materials and methods

Protein retrieval

The CELLO v.2.5: Sub-cellular localization predictor tool (http://cello.life.nctu.edu.tw/) was used to estimate the subcellular location of the sequence AsaA.

Antigenic and allergenic predictions

The score for the antigenicity of AsaA was predicted by applying the VaXiJen v2.0 server (https://www.ddg-pharmfac.net/vaxijen/). Antigens are categorized based on the physical and chemical properties of the proteins and are based on the prediction model of the protein alignment [12]. Allergenicity of the AsaA is predicted using the AlgPred server (http://crdd.osdd.net/raghava/algpred/) which is employed to predict the proteins that are allergenic based on the mapping of immunoglobulin E (IgE) epitopes.

Secondary structure prediction

The secondary structure of the AsaA was predicted using the Self-Optimized Prediction Method with Alignment (SOPMA) server (https://npsa-prabi.ibcp.fr/cgi-bin/npsa_automat.pl?page=/NPSA/npsa_sopma.html). Predictions were made for the amount of beta-sheet, turns, and coils.

Predictions of T-cell immunodominant epitopes

T-cell immunodominant epitopes restricted to MHC class II molecules are crucial for the start of the immune response that is directed against an antigen. The EpiDOCK tool (https://www.ddg-pharmfac.net/epidock/EpiDockPage.html) was applied to predict the T-cell epitopes of AsaA, to check for the binding of epitopes to 12, 6, and 5 HLA alleles respective to -DR, -DQ, and -DP alleles. The query sequence was given as input in the FASTA format and the tool was applied to detect the maximum number of binders for the predicted epitopes for the best assessments.

Predictions on stable and antigenic T-cell epitopes

The VaXiJen 2.0 server was used to predict antigenicity, and the ProtParam server (https://web.expasy.org/protparam/) was used to determine the instability index of predicted epitopes.

Predicted epitopes structure prediction and validation

The PEP-FOLD server (https://mobyle.rpbs.univ-paris-diderot.fr/cgi-bin/portal.py#forms::PEP-FOLD) is a de novo approach used to identify the structures of the peptide from amino acid sequences and further employed to detect the MHC class II binders. Using the RAMPAGE program (https://bio.tools/RAMPAGE), which forecasts the stereochemical characteristics of a given structure, the projected structure was verified. By forecasting which amino acids will lie in the preferred, permitted, and disallowed sections of the Ramachandran plot, the server evaluates the quality of simulated epitopes.

Interactions of the selected epitopes with HLA alleles

From the protein data bank (PDB) database, the three-dimensional structures of the HLA alleles specific to -DP, -DQ, -DR, and TLR-2 were retrieved as 3LQZ, 5KSV, 4AH2, and -6NIG, respectively. Using the ClusterPro tool (https://cluspro.org/help.php), the molecular interactions of the binders of MHC with HLA-alleles were performed.

B-cell epitope prediction

Using the Kolaskar and Tongaonkar Antigenicity Prediction technique (http://tools.iedb.org/bcell/), the Immune Epitope Database (IEDB) (https://www.iedb.org/) was used to predict the AsaA B-cell epitope.

## Results

Protein retrieval

The AsaA sequence from *A. baumannii *was observed with the sequence ID R9RHI2 in the database of the UniProt (Universal Protein Resource) (https://www.uniprot.org/). The subcellular location of the predicted protein was assessed to be at the periplasmic membrane (2.865*) using CELLO v.2.5: subCELlularLOcalization predictor tool (http://cello.life.nctu.edu.tw/).

Antigenicity and allergenicity results

The antigenicity of AsaA was predicted based on the antigenicity score obtained from the VaXiJen v2.0 server and it was observed to be 0.5478. The epitope was observed to be non-allergen in the AlgPred tool that is obtained by the support vector machine (SVM) based method relying on the mapping of epitope-specific to IgE (Table 1).

**Table 1:** Identification of major histocompatibility class II (MHC class II) binders (T-cell epitopes) and prediction of antigenicity and stability prediction of selected T-cell epitopes using VaxiJen and ProtParam HLA: human leukocyte antigen

Position of peptide	Sequence	Number of binders to HLA alleles (DP, DQ, DR)	VaxiJen v 2.0	ProtParam
1	MLKQSVLIL	14	-0.4152	95.99
2	LKQSVLILF	12	0.1329	95.99
3	KQSVLILFL	14	0.6194	105.42
7	LILFLIGNY	15	0.6852	20.86
12	IGNYVGLSC	11	0.5946	26.24
15	YVGLSCAQA	13	1.1138	35.68
18	LSCAQAAEL	15	0.9975	45.11
29	IKLKKSCIK	18	1.2485	7.38
30	KLKKSCIKK	17	0.9433	16.81
33	KSCIKKNPI	13	0.5314	32.48
36	IKKNPIIEG	10	0.2056	45.08
41	IIEGQTDPE	10	0.2272	77.02
50	LLNLFKQVC	10	-0.3243	10.12
51	LNLFKQVCD	12	0.3469	31.52

Protein secondary structure

**Figure 1:**
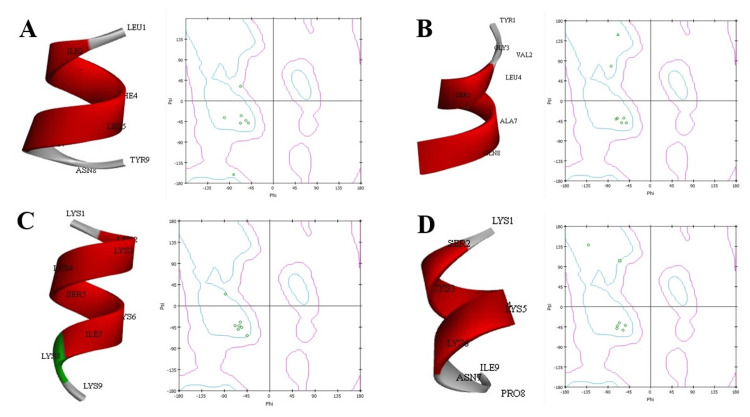
Structure predictions of the predicted epitopes using PEP-FOLD server and its validation by Ramachandran plot: A. LILFLIGNY (LEU1, ILE2, PHE4, LEU6, TYR 9, ASN8); B. (TYR1, GLY3, VAL2, LEU4, SER3, ALA7, GLN8); C. YVGLSCAQA (LYS1, LYS2, LYS3, LYS4, SER5, LYS6, ILE7, LYS8, LYS9); D. KLKKSCIKK (LYS1, SER2, LYS3, LYS5, LYS6, ILE9, ASN9, PRO8)

*A. baumannii *depicted a secondary structure representing an alpha helix of 43.81%, an extended strand of 7.52%, a beta-turn of 2.65%, and a random coil of 46.02% (Figure 1).

Antigenicity and stability results

The peptide binders that were ≥10 were selected for the evaluations of antigenic and further to evaluate its stability. The peptides showing an antigenicity score of >0.4 were assessed as antigenic epitopes. Similarly, the epitopes were categorized as stable based on the ProtParam scores <40. From the final 20 peptides predicted, 14 peptides were observed as antigenic, while the remaining six were observed as stable peptides (Table 2).

**Table 2:** Structure prediction using PEP-FOLD and Ramachandran plot evaluation showing the epitopes at the most favorable region HLA: human leukocyte antigen

Position of peptide	Sequence	Number of binders to HLA alleles (DP, DQ, DR)	VaXiJen v 2.0	ProtParam
1	MLKQSVLIL	14	-0.4152	95.99
2	LKQSVLILF	12	0.1329	95.99
3	KQSVLILFL	14	0.6194	105.42
7	LILFLIGNY	15	0.6852	20.86
12	IGNYVGLSC	11	0.5946	26.24
15	YVGLSCAQA	13	1.1138	35.68
18	LSCAQAAEL	15	0.9975	45.11
29	IKLKKSCIK	18	1.2485	7.38
30	KLKKSCIKK	17	0.9433	16.81
33	KSCIKKNPI	13	0.5314	32.48
36	IKKNPIIEG	10	0.2056	45.08
41	IIEGQTDPE	10	0.2272	77.02
50	LLNLFKQVC	10	-0.3243	10.12
51	LNLFKQVCD	12	0.3469	31.52

Results of epitope structure predictions 

The selected proteins were subjected to the structure predictions in the PEP-FOLD server, and the structures were validated using the Ramachandran plot analysis. Based on the obtained results, six epitope peptides out of the 18 were short-listed for the final docking interactions, along with HLA restriction analysis (Table 3).

**Table 3:** Molecular docking scores upon the interaction of the predicted epitopes with HLA-alleles and TLR-2 using the PEP-FOLD server and its validation by Ramachandran plot HLA: human leukocyte antigens; TLR-2: toll-like receptor 2

Predicted peptides	Peptides	Most favored region
Epitope 1	LILFLIGNY	100%
Epitope 2	YVGLSCAQA	100%
Epitope 3	KLKKSCIKK	100%
Epitope 4	KSCIKKNPI	100%
Epitope 5	IGNYVGLSC	42.8%
Epitope 6	IKLKKSCIK	42.8%

Results of docking interactions of the epitopes with HLA alleles 

**Figure 2:**
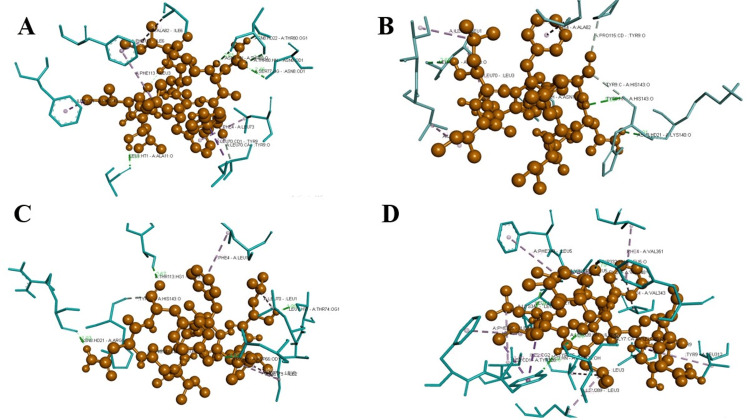
Docking interactions of the epitope LILFLIGNY with A. HLA-DP, B. HLA-DQ, C. HLA-DR, and D. TLR-2 using VaxiJen and ProtParam HLA: human leukocyte antigen; TLR-2: toll-like receptor 2

Molecular docking interactions of the selected peptides with the HLA-alleles were successful and the final epitope LILFLIGNY was selected as the best immune-dominant epitope with good docking interaction scores (Table 4) possessing high affinity yielding the maximum number of hydrogen bonds (Figure 2).

**Table 4:** Molecular docking scores upon the interaction of the predicted epitopes with HLA-alleles and TLR-2 HLA: human leukocyte antigen; TLR-2: toll-like receptor 2

Molecular docking of epitopes with HLA-alleles	Epitopes	HLA-DP	HLA-DQ	HLA-DR	TLR-2
Epitope 1	LILFLIGNY	-922.9	-830.5	-807.8	-1075.4
Epitope 2	YVGLSCAQA	-643.3	-648.7	-685.7	-934.3
Epitope 3	KLKKSCIKK	-622.8	-563.3	-623.2	-554.1
Epitope 4	KSCIKKNPI	-625.5	-569.3	-637.3	-738.2

B-cell antigenicity predictions

**Figure 3:**
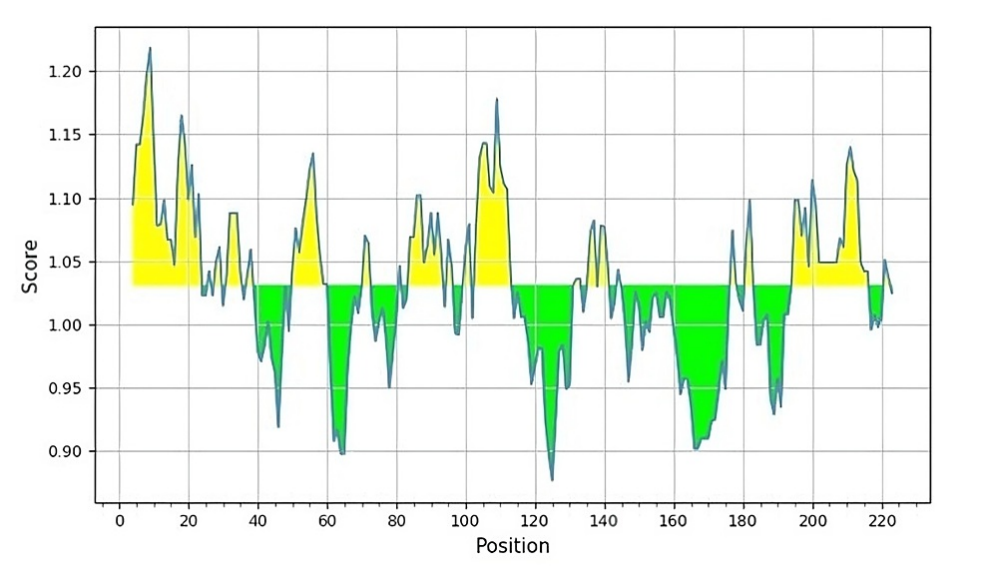
Kolaskar and Tongaonkar antigenicity predictions of the B-cell dominant LILFLIGNY epitope The X-axis and Y-axis denote the sequence position and antigenic propensity, respectively The regions above the threshold value are antigenic and are depicted in yellow

Predictions of the B-cell antigenicity for the epitope LILFLIGNY assessed using the Kolaskar & Tongaonkar tool were promising. The X-axis and Y-axis were checked for the position of the amino acid sequence and the propensity of the antigen, respectively. Antigenic positions were observed based on the threshold value that appeared in yellow and considered antigenic (Figure 3).

## Discussion

The present study is based on the synthesis of peptide vaccine from the immune-dominant epitopes from *A. baumannii* infections that are common and linked to high morbidity, death, and multi-resistance rates. Acquired MDR, which makes it difficult to find effective antimicrobials, is the biggest issue in complicating the treatment of recalcitrant infections [12]. MDR can result from the presence of a single resistance determinant, such as an MDR pump, which confers resistance to multiple classes of antimicrobial agents, particularly in patients with co-morbidities. Recently, it has also emerged as an oral and dental pathogen [13-14]. Many resistance genetic determinants are associated with the propensity of MDR [15-16]. The T6SS plays a vital role in the anti-phagocytic process and thus attributes virulence to* A. baumannii.* In the present investigation, the AsaA T6SS was selected for the identification of B-cell and T-cell dominant epitope peptides, which will be utilized in further vaccine construct development. In-silico-based predictions aid in the preliminary assessment of the cell-mediated and humoral immune responses.

Cytoplasmic membrane proteins play a vital role in developing small molecule drugs while the membrane peptides are utilized for the formation of vaccines. The location predictions of the AsaA secretion systems have shown the periplasmic spaces and are promising for the design of the peptides. The antigenic epitopes of a few potent virulence proteins were done for designing the vaccine against *A. baumannii *using computational-based tools and databases in many earlier studies. Antigenicity and allergenicity predictions were also promising for the selection of the epitopes for further evaluation. The selected peptide binders (>10) subjected to the evaluation of their antigenic, and stability properties were assessed based on the default set score >0.4 in the VaXiJen prediction tool. An initial selection of 18 peptides was made from a total of 20 peptides for further analysis. Allergenicity was assessed by the SVM method using the prediction analysis based on the mapping of the IgE epitope.

The PEP-FOLD server was used for the prediction of the peptide structures for the epitopes and was validated using the RAMPAGE tool for assessing the stereo-chemical properties of the structures. Based on the PEP-FOLD server evaluation, around 10 epitopes were shortlisted for further structural validation. From the predicted epitopes, four epitopes were in the favored regions by the Ramachandran validation. The designed vaccine binding affinity was further evaluated by molecular docking to six variable HLA molecules, and the epitope LILFLIGNY showed the highest interactions with good docking scores as -830.5, -807.8, and -1075.4 for HLA-DQ, DR, and TLR-2, respectively. Conventional hydrogen bonds, hydrophobic interactions, alkyl-pi, and pi-pi stacks were also in a higher state suggesting the increase in the stability and strength of a vaccine-receptor interaction sustainability. Similar studies also have proved that such in-silico analysis and vaccine predictions are cheaper, and one of the most efficient methodologies in the field of drug and vaccine discovery [17-18]. Developing vaccines for MDR strains is a novel approach to effectively address the antimicrobial resistance (AMR) traits. The limitations of this in-silico analysis conducted on the LILFLIGNY epitope stride lacked experimental validation. Additionally, protein detection in in-vitro studies and further analysis, as well as preclinical trials (animal studies), are necessary steps for synthesizing vaccines from immune-dominant epitope peptides. Looking ahead, clinical studies on this vaccine targeting MDR traits and enhancing immune response through B-cell and T-cell activation hold promise for addressing the AMR challenge.

## Conclusions

In the present investigation, it was found that epitope LILFLIGNY is a promising immune-dominant peptide to evoke host immune responses. Computational platforms are promising in the design of immune-dominant peptides and for further vaccine synthesis. The findings of the present study also warrant the need for further experimental evidence-based studies on its toxicity and immunological memory. The present investigation concludes with LILFLIGNY as a suitable epitope vaccine peptide that may be a candidate to combat the complications of the infections caused by *A. baumannii.*
